# The Course Of IGF-1 Levels and Nutrient Intake in Extremely and Very Preterm Infants During Hospitalisation

**DOI:** 10.3390/nu12030675

**Published:** 2020-03-02

**Authors:** Dana F.J. Yumani, Alexandra K. Calor, Mirjam. M. van Weissenbruch

**Affiliations:** Amsterdam UMC, Department of Pediatrics, VU University Medical Center, 1081 HV Amsterdam, The Netherlandsm.vanweissenbruch@amsterdamumc.nl (M.M.v.W.)

**Keywords:** preterm infants, insulin-like growth factor, nutrient intake, postnatal growth

## Abstract

Background: Insulin-like growth factor 1 (IGF-1) plays an important role in the complex association between nutrition, growth, and maturation in extremely and very preterm infants. Nevertheless, in this population, research on associations between IGF-1 and nutrition is limited. Therefore this study aimed to evaluate the possible associations between the course of IGF-1 levels and nutrient intake between preterm birth and 36 weeks postmenstrual age (PMA). Methods: 87 infants born between 24 and 32 weeks gestational age were followed up to 36 weeks PMA. Actual daily macronutrient intake was calculated, and growth was assessed weekly. IGF-1 was sampled from umbilical cord blood at birth and every other week thereafter. Results: There was an inverse relationship between the amount of parenteral nutrition in the second week of life and IGF-1. Total protein, fat, and carbohydrate intake, as well as total energy intake, primarily showed a positive association with IGF-1 levels, particularly between 30 and 33 weeks PMA. Gestational age, bronchopulmonary dysplasia (BPD), and weight were significant confounders in the association between nutrient intake and IGF-1 levels. Conclusion: Parenteral nutrition was found to be a negative predictor of IGF-1 levels, and there could potentially be a time frame in which macronutrient intake is unable to impact IGF-1 levels. Future research should aim to narrow down this time frame and to gain more insight into factors enhancing or decreasing the response of IGF-1 to nutrition, e.g., age and inflammatory state, to align nutritional interventions accordingly.

## 1. Introduction

Preterm birth leads to an abrupt disruption of fetal development, leaving preterm infants in a precarious situation where they need to thrive despite an immature gastrointestinal tract and not fully developed immune and endocrine functions. Insulin-like growth factor 1 (IGF-1) stimulates growth and plays a crucial role in the complex association between early nutrient intake, growth, and maturation [1]. In preterm infants, IGF-1 is mainly stimulated by insulin and nutrition [1,2]. However, to what extent various macronutrients impact IGF-1 levels in different phases of postnatal life is yet to be elucidated.

In the few studies relating actual nutrient intake to IGF-1 levels between preterm birth and hospital discharge, protein and energy intake had a positive association with IGF-1 levels [3,4,5]. Remarkably, one previous study reported that in preterm infants, the positive association between IGF-1 and nutrient intake was only apparent after 30 weeks postmenstrual age (PMA) [4]. This suggests that there might be a limited window of opportunity for nutrition to influence early postnatal growth.

It is to be noted that, to the best of our knowledge, only protein and energy intake have been studied in relation to IGF-1 levels in preterm infants. Interestingly, in adults, studies assessing fat and carbohydrate intake in relation to IGF-1 have been inconclusive. This leaves us with a gap in knowledge concerning the potential impact of dietary fat and carbohydrate intake on IGF-1 levels in preterm infants [6,7,8].

In addition, the route of nutrient administration is another largely uncharted research area in relation to IGF-1 levels in preterm infants. Animal studies have shown that in a state of inflammation or poor nutrient intake, enteral feeding results in higher IGF-1 levels than parenteral feeding. This is thought to be due to a reduction in inflammatory cytokine levels after enteral feeding [9,10]. These findings suggest that the route of nutrient administration could mediate cytokine production and consequently influence IGF-1 levels. To our knowledge, this is yet to be investigated in preterm infants.

Given the impact of poor growth and subsequent accelerated growth on later health outcomes in infants born preterm [11,12,13], it is pertinent to gain insight into factors influencing early postnatal growth, in order to obtain potential interventions to avert later adverse outcomes. In this light, the association between nutrition and IGF-1 is of particular interest, because nutrition is a factor which lends itself well for intervention and could lead to changes in clinical practice. Nevertheless, research on the relationship between nutrition and IGF-1 in preterm infants is scarce, and most studies were published over a decade ago. Meanwhile, nutrition and neonatal intensive care have significantly changed. In addition, in previous studies, the infants were either on full enteral feeds or the relationship with the proportion of parenteral feeding was not taken into account. In this explorative observational study, associations between the macronutrient intake, the proportion of parenteral feeding, and IGF-1 were assessed in very and extremely preterm infants between birth and 36 weeks PMA.

## 2. Methods

### 2.1. Study Population

This paper describes the results of the “Nutrition in relation to the endocrine regulation of preterm growth” (NUTRIE) study, a longitudinal observational study on nutrition in relation to the endocrine regulation of growth and body composition in preterm infants. Eighty-seven participants were enrolled between September 2015 and July 2018. Infants born between 24 and 32 weeks of gestation were eligible for study participation if they were born without substantial congenital anomalies, and were admitted to the neonatal intensive care unit (NICU) of Amsterdam UMC, location VU University Medical Center in Amsterdam, The Netherlands. Informed consent was obtained in the first week of life. The study was approved by the medical ethics committee of the VU Amsterdam and was registered at the Dutch Trial Register (www.trialregister.nl; NTR5311).

### 2.2. Nutrition

Infants initially received total parenteral nutrition and minimal enteral feeding. During total parental feeding, clinicians aimed to achieve an energy intake of 85–100 kcal kg^−1^ day^−1^, a protein intake of 3–4 g kg^−1^ day^−1^, and a fat intake of 3–3.5 g kg^−1^ day^−1^. One the first day of life, parenteral glucose administration was targeted at 5.5–7 mg.kg^−1^ min^−1^, going up to maximum 12 mg kg^−1^ min^−1^ after the first week of life, depending on blood glucose levels. Full enteral feeding (160 mL kg^−1^ day^−1^) was aimed to be achieved within 7 to 10 days after birth with a total protein intake of 3.5 to 4.5 g kg^−1^ day^−1^ and a total energy intake of 110 to 140 kcal kg^−1^ day^−1^. Infants were primarily fed human milk. If own mother’s milk was insufficient or unavailable, donor human milk was administered up to 32 weeks PMA, followed by preterm starters formula until discharge home. If parents declined the use of donor human milk, infants were fed preterm starters formula from birth, whenever own mother’s milk was unavailable.

Clinicians aimed to achieve 15–20 g weight gain kg^−1^ day^−1^, with a weight SD score above −1 SD. Head circumference growth was targeted at 1 cm per week and length at 1.25 cm per week.

Breast milk fortifier (Nutrilon Nenatal Breast Milk Fortifier, Nutricia, Wageningen, The Netherlands) was added to human milk once an enteral intake of 100 mL kg^−1^ day^−1^ was achieved. In case of poor growth, as assessed by the clinician in charge, intake was increased to a maximum of 180 mL/kg, permitted that the infant’s condition allowed for an increased fluid intake. If poor growth persisted, up to 1% protein fortifier (Nutrilon Nenatal Protein Fortifier, Nutricia, Wageningen, The Netherlands) was added to the fortified human milk. Lastly, up to 4% of a high-energy, long-chain triglyceride, fat emulsion (Calogen, Nutricia, Wageningen, The Netherlands) was added if growth remained restricted despite fortification. In case of growth restriction in formula-fed infants, intake was increased to 180 mL kg^−1^ day^−1^ and an additional 1.5 g of preterm starters formula was added per 100 mL formula (Nutrilon Nenatal Start, Nutricia, Wageningen, The Netherlands). In addition, protein fortifier and a fat emulsion could be added to the formula if poor growth persisted.

### 2.3. Study Procedures

All participants were admitted to the NICU of Amsterdam UMC, location VU University medical center within 24 h from birth. Infants in good clinical condition were discharged to step-down units in other hospitals at a PMA of 30 weeks and a weight of at least 1000 g.

Obstetric data, clinical condition and intake up to 36 weeks PMA were collected from hospital records.

### 2.4. Growth

Growth was assessed weekly between birth and 36 weeks PMA. Weight was measured on an electronic scale to the nearest gram, length was measured on a length board to the nearest 0.1 cm, and occipital-frontal head circumference was measured with a nonstretchable measuring tape to the nearest 0.1 cm. The measurements were done by the nursing staff.

Standard deviation scores (SDS) of weight, length and head circumferences were calculated according to Fenton [14].

### 2.5. Intake

Daily macronutrient intake was calculated from actual intake data obtained from hospital records. Own mother’s milk composition was based on reference values [15,16] (Table 1). Donor human milk composition was based on analyses of the donor milk batches administered to the first 23 study participants.

### 2.6. Endocrine Parameters

IGF-1 was sampled from umbilical cord blood at birth and from venipuncture or capillary puncture every other week between birth and 36 weeks PMA. A chemiluminescence immunoassay (LIAISON^®^, DiaSorin, Saluggia, Italy) was used to analyze IGF-1 (intra-assay percent coefficient of variation (% CV): 8%, inter-assay % CV: 7%). The number of samples per week PMA is depicted below (Table 2).

### 2.7. Potential Confounders

The following comorbidities were assessed as potential confounders in the association between nutrient intake and IGF-1:

- Bronchopulmonary dysplasia (BPD); defined as having had a need for supplemental oxygen for at least 28 days at 36 weeks PMA or discharge home (whichever came first) [17].
- Necrotizing enterocolitis (NEC); classified according to the Modified Bell’s staging criteria [18].
- Late-onset sepsis (LOS), defined as sepsis occurring 72 h after birth with a positive blood culture or a full course of antibiotic treatment [19].
- Retinopathy of prematurity (ROP), classified according to the International Classification for Retinopathy of Prematurity [20].
- Intraventricular hemorrhage (IVH), classified according to the Papile grading system [21].
- Patent ductus arteriosus (PDA), which was defined as hemodynamically significant if treatment was prescribed [22].

In addition, gender, gestational age at birth, postmenstrual age at the time of blood sampling, weight and weight SD score were assessed as potential confounders.

### 2.8. Statistical Analysis

The change in IGF-1 over time was predicted for each individual using mixed models. The associations between nutrient intake, IGF-1, and potential confounders were assessed with regression analyses. Analyses were conducted using IBM^®^ SPSS^®^ Statistics 26 for Windows (IBM Corp., Armonk, NY, USA). Two-sided statistical significance was assumed at *p*-values less than 0.05.

## 3. Results

**Figure 1:**
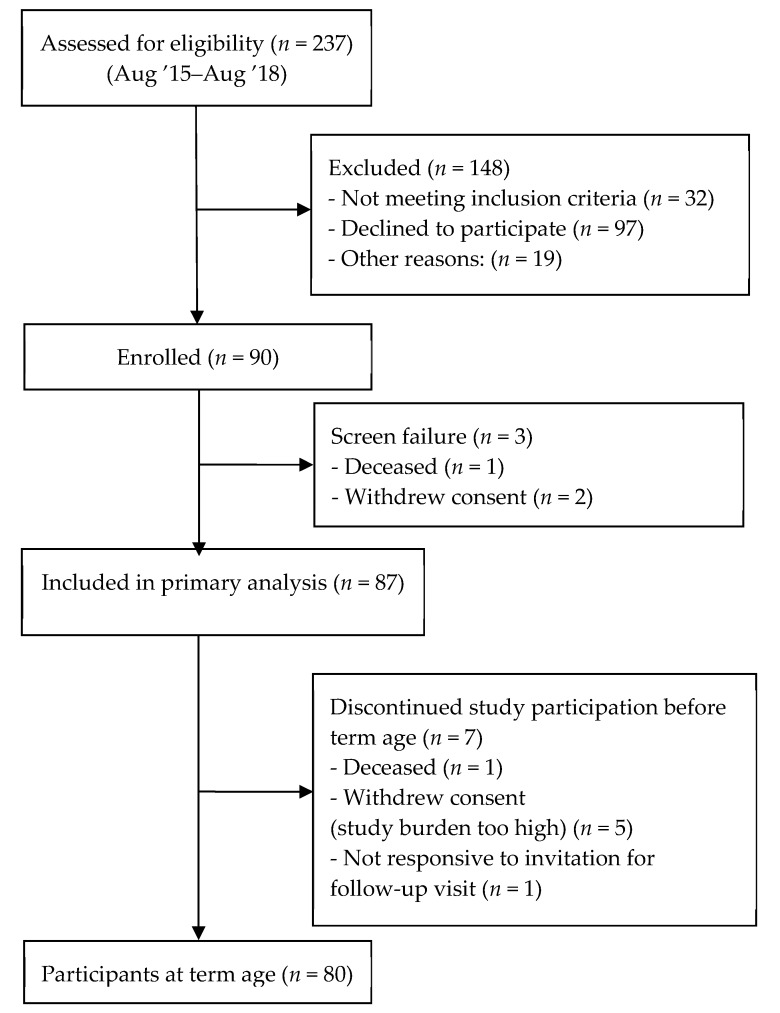
Flow diagram of participants included in the study.

Eighty-seven infants were included in primary analysis (Figure 1). Baseline characteristics are shown in Table 3.

### 3.1. Changes in IGF-1 During Hospitalisation

**Figure 2:**
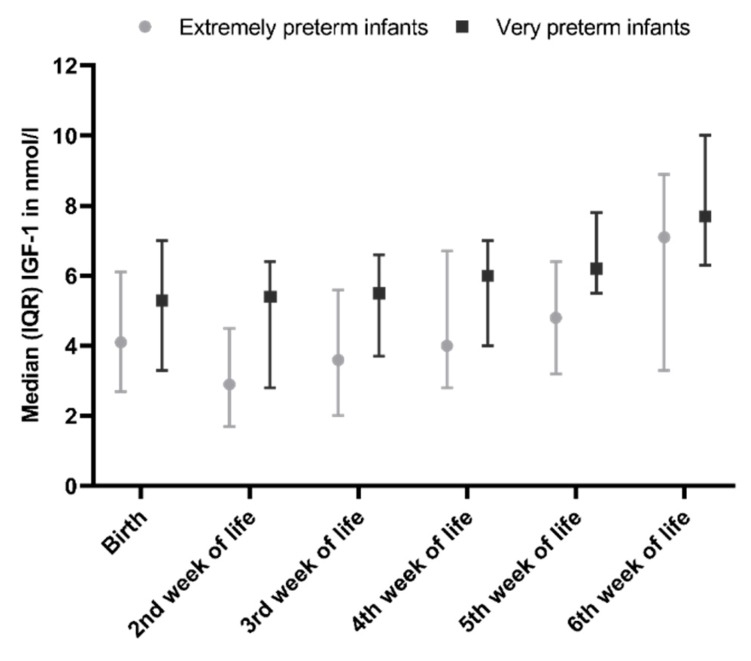
Insulin-like growth factor 1 (IGF-1) levels in extremely preterm and very preterm infants.

Between birth and the second week of life, IGF-1 levels dropped from 4.8 nmol/L to 3.2 nmol/L in extremely preterm infants (mean decrease −1.5, 95% CI −5.2–2.2, *p* = 0.314). In very preterm infants, IGF-1 showed a mean decrease of 0.3 nmol/L (95% CI −2.1–1.4, *p* = 0.675) between birth and the second week of life. From the second week of life, IGF-1 showed a mean (SD) increase of 0.6 (0.2) nmol/L per week in very preterm infants and 0.7 (0.1) nmol/L per week in extremely preterm infants (mean difference 0.1, 95% CI 0.0–0.2, *p* = 0.143) (Figure 2).

Compared to boys, at birth, IGF-1 levels were lower in girls. In addition, IGF-1 levels had an inverse relationship with gestational age at birth and PMA at the time of sampling. Postnatal age in days at the time of sampling did not predict IGF-1 levels. After correcting for weight, IGF-1 levels were no longer predicted by gender, gestational age at birth, and postmenstrual age at the time of blood sampling either, and weight remained the only significant predictor of IGF-1 levels.

### 3.2. IGF-1 Levels in Relation to Growth

**Figure 3:**
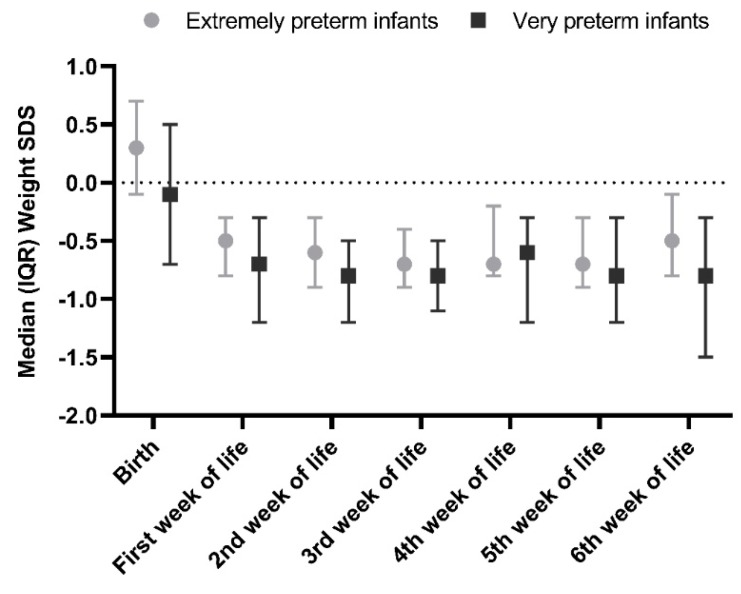
Weight SD score in extremely and very preterm infants.

Mean birth weight SDS was 0.04, with three out of 87 infants being small for gestational age (weight SDS < −1.3) (Figure 3). At 36 weeks PMA 17 out of 80 infants had a weight SDS below −1.3 SDS. Between the second week of life and 36 weeks PMA, five out of 80 infants showed catch-up growth (increase in weight SDS > 0.67). IGF-1 positively correlated with previous, concurrent, and subsequent weight and weight SDS. When weight SDS was corrected for absolute weight in grams, only weight remained a significant predictor of IGF-1 levels. Compared to infants with a weight of 1000 g or more, infants with a weight below 1000 g had a 2.5 nmol/L lower IGF-1 at two weeks postnatal age (95% CI −3.7–−1.3, *p* < 0.001).

### 3.3. IGF-1 Levels and Route of Administration

**Figure 4:**
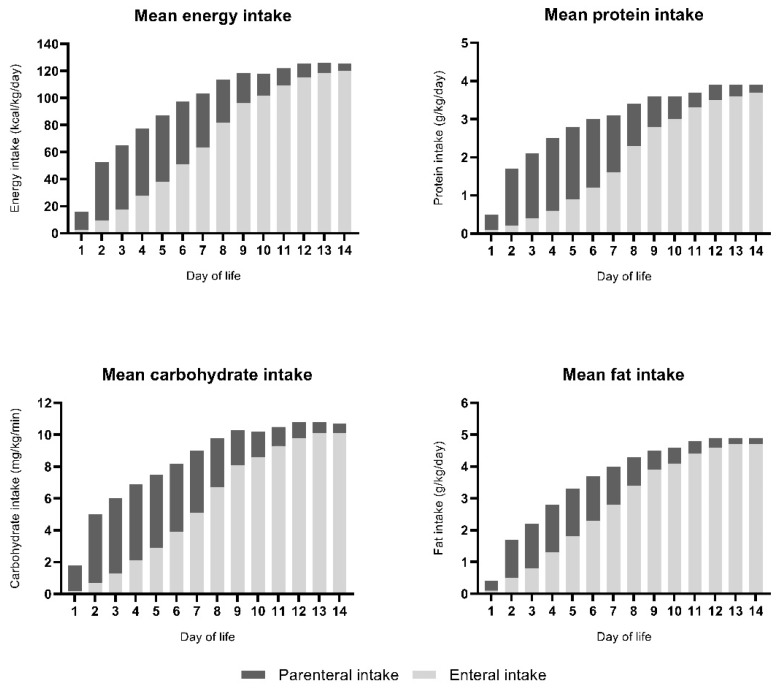
Parenteral and enteral nutrient intake in preterm infants in the first two weeks of life.

**Figure 5:**
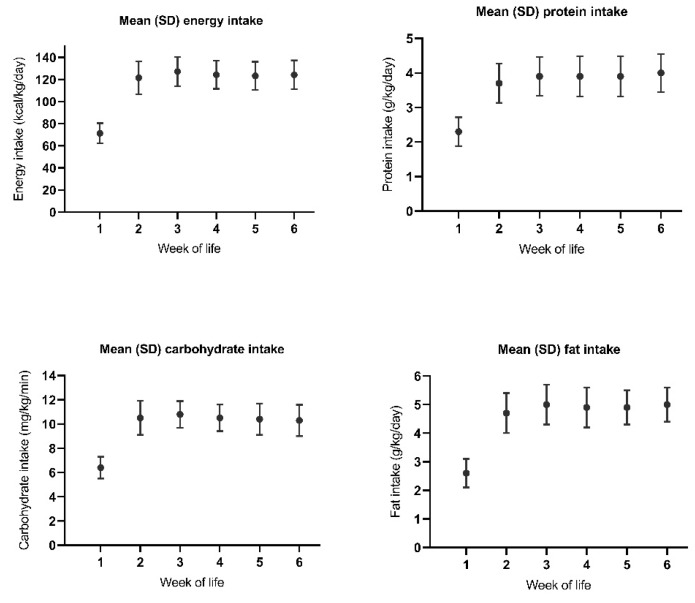
Nutrient intake in preterm infants in the first six weeks of life.

On the fourteenth day of life, 80% of infants received full enteral feeding. Figure 4 displays the ratio between parenteral and enteral intake in the first two weeks of life. At two weeks postnatal age, 73 out of 87 infants were fed more than 90% own mother’s milk, 10 were fed donor human milk, and 4 were formula-fed. From the second week of life, mean nutrient intake was within the references of our local protocol (Figure 5). The percentage of parenteral intake from the eighth through to the twelfth day of life, expressed as $\frac{\mathrm{parenteral}\;\mathrm{energy}\;\mathrm{intake}}{\mathrm{total}\;\mathrm{energy}\;\mathrm{intake}}$, was associated with lower IGF-1 levels at two weeks postnatal age. Gestational age, weight, BPD, and hemodynamically significant PDA were confounders in the relationship between parenteral intake and IGF-1 levels. Based on the F-change, the best predictive model included weight, BPD, and hemodynamically significant PDA. After correcting for these confounders, the association between the percentage of parenteral nutrition and IGF-1 levels remained significant (Table 4).

Day 1 is not equal to 24 h for all study subjects.

### 3.4. Nutrient Intake in Relation to Concurrent IGF-1 Levels

Positive associations were found between energy intake and IGF-1 levels at 30 to 33 weeks PMA (Table 5). BPD was a significant confounder from 32 weeks PMA. Protein, carbohydrate, and fat intake showed a similar pattern (Table 5). In addition, however, protein intake showed a positive association with IGF-1 levels at 28 weeks PMA: per gram increase in protein intake IGF-1 levels showed an increase of 1.1 nmol/L, R² = 0.506, *p* = 0.032. This is in contrast to a lack of associations at 29 weeks PMA with a larger sample size (*n* = 12) than the sample size at 28 weeks PMA. At 28 weeks PMA, IGF-1 was measured in six infants, of whom five had a recent history of sepsis and required an erythrocyte transfusion within 24 h of the blood sampling. Nutrient intake per kg body weight was not associated with IGF-1 levels at any point in time. After correcting for weight in multivariate analysis, the associations between total nutrient intake and IGF-1 lost their significance (Table 5). In univariate analysis at 30 weeks PMA, weight explained 45% of the variance in IGF-1 levels, compared to 33% of the variance that was explained by nutrient intake. By 33 weeks PMA, these numbers declined to respectively 17% and 15%.

### 3.5. Nutrition in Relation to Changes in IGF-1 According to Postnatal Age

The change in IGF-1 levels in the first four weeks of life was positively associated with protein, carbohydrate, fat, and total energy intake (after correction for gestational age). IGF-1 levels increased with 0.01 nmol/L per 10 kcal, R² = 0.266, *p* < 0.001. Comorbidities were not a significant confounder. After correcting for weight, total nutrient intake was no longer a significant predictor of change in IGF-1.

### 3.6. Nutrition in Relation to Changes in IGF-1 According to Postmenstrual Age

Looking at postmenstrual age, there was a positive association between total nutrient intake from 28 through 31 weeks PMA and the change in IGF-1 between birth and 32 weeks PMA (after correcting for gestational age). IGF-1 levels increased with 0.2 nmol/L per 10 kcal, R² = 0.287, *p* = 0.002. Comorbidities were not a significant confounder. However, after correcting for weight, energy intake could no longer predict the change in IGF-1. All macronutrients showed a similar pattern.

## 4. Discussion

This study shows that the proportion of parenteral nutrition is negatively associated with IGF-1 levels in extremely and very preterm infants. Gestational age, BPD, and weight were significant confounders in the association between nutrient intake and IGF-1 levels. Total protein, fat, and carbohydrate intake, as well as total energy intake, showed a positive association with IGF-1 levels, particularly between 30 and 33 weeks PMA.

### 4.1. The Effect of the Various Macronutrients on IGF-1 Levels

Studies in preterm infants consistently show that protein intake is positively associated with IGF-1 levels [3,4,5]. However, not all studies could show that energy intake was a predictor of IGF-1 after correction for confounders [3]. In our study, higher total protein intake and higher total energy intake were associated with higher IGF-1 levels. In contrast to previous studies, our study did not find an association between nutrient intake per kg bodyweight and IGF-1 levels. Weight explained more of the variance in IGF-1 than nutrient intake. However, it should be noted that the variance in nutrient intake per kg bodyweight may have been too small to show significant differences in IGF-1 levels in our population. This is due to the univocal application of our local nutrition protocol. For example, from 33 weeks PMA the interquartile range in protein intake was between 3.7 and 4.1 g kg^−1^ day^−1^. This range was notably smaller compared to previous research [4] and could have limited the statistical power.

To our knowledge, the impact of fat and carbohydrate intake on IGF-1 levels in preterm infants has not been studied previously. Meanwhile, studies in adults have been inconclusive, with some finding positive associations [7], while others found negative associations [6] or no association at all [8]. In our study, total fat and total carbohydrate intake showed a positive association with IGF-1 levels. Interestingly, both carbohydrate and fat intake had a comparable impact on IGF-1 when compared to protein intake—a one SD change in any of the macronutrients led to a change of 0.6 SD in IGF-1 levels at 30 weeks PMA. Although it has been suggested that the role of proteins is more important than that of carbohydrates and fat in stimulating IGF-1 [3,6], like dietary proteins, dietary fat and carbohydrates have been found to increase hepatic IGF-1 expression in animal studies [23]. Moreover, fat and carbohydrates provide the majority of the total energy intake, which has repeatedly been shown to have a positive association with IGF-1 levels and thus supports our findings.

### 4.2. The Route of Nutrient Administration

Parenteral feeding was found to be negatively associated with IGF-1 levels. It could be hypothesized that less exposure of the gastrointestinal tract to nutrition enhances a pro-inflammatory state in the immature gut, which in turn could lead to lower IGF-1 levels. Indeed, a pro-inflammatory state in preterm infants has been associated with decreased IGF-1 levels [24]. It has also been demonstrated that colostrum and maternal milk contain high concentrations of anti-inflammatory cytokines [25]. These anti-inflammatory cytokines could potentially lower the relatively pro-inflammatory state in the immature gut and consequentially increase IGF-1 levels. In one study, preterm infants who received own mother’s milk from birth were shown to have higher levels of IGF-1 at term equivalent age compared to those who were formula fed from birth [26]. Moreover, animal studies found that in a state of inflammation or nutrient deprivation, parenteral feeding was associated with lower IGF-1 levels compared to enteral feeding. This appears to be due to a decrease in pro-inflammatory cytokines after enteral feeding [9,10]. This leads us to believe that the neutralizing effect of anti-inflammatory cytokines, which are particularly abundant in colostrum and breast milk, is diminished and results in lower IGF-1 levels when parenteral nutrition is increased.

It could also be suggested that infants who received relatively higher proportions of parenteral nutrition were the more vulnerable, smaller, younger, and iller infants, and thus the association with lower IGF-1 levels. However, after correcting for gestational age, weight, and comorbidities, parenteral nutrition remained a significant predictor of IGF-1 levels.

### 4.3. Window of Effect of Nutrient Intake on IGF-1 Levels

In our population, the influence of nutrition on IGF-1 levels seemed to be most apparent between 30 and 33 weeks PMA. Hypothesizing, preterm infants may have to reach a certain level of maturity before an impact of nutrition on the IGF-1 axis can be noted. In support of this, Smith and colleagues found that the magnitude of the rise in IGF-1 levels per gram protein increased with increasing gestational and postnatal age. Hansen-Pupp and colleagues also found nutrient intake not to influence IGF-1 levels at lower postmenstrual ages, but only from 32 weeks PMA onwards. Speculatively, other factors than maturity could influence when IGF-1 levels start to respond to nutrient intake. Of note, in our study, in a set of infants who were ill, a positive association between total protein intake and IGF-1 levels was already found at 28 weeks PMA. This was in contrast to the other macronutrients and total energy intake, which only showed positive associations with IGF-1 levels from 30 weeks PMA onwards. Among the set of infants of whom blood was sampled at 28 weeks, all but one had a recent history of sepsis and anemia requiring erythrocyte transfusion. It could be speculated that the state of inflammation triggered a higher sensibility of the IGF-1 axis to protein uptake. Despite their IGF-1 levels still being low, 1 g of protein might have triggered more increase in IGF-1 compared to infants who were not ill.

It is noteworthy that in our population, no associations between nutrient intake and IGF-1 levels were found at 34 and 35 weeks PMA, in contrast to other studies [3,4,5]. However, Ëngstrom and colleagues found that in infants with a weight of less than 2000 g, protein supplementation had a stronger association with IGF-1 levels compared to infants over 2000 g. Perhaps this can explain why the positive trend our study found at 34 and 35 weeks PMA was not statistically significant.

In contrast to the relationship between nutrient intake and concurrent IGF-1 levels described above, our results showed nutrient intake to influence the change in IGF-1 at a younger PMA. For every macronutrient, intake from 28 weeks PMA was associated with the change in IGF-1 levels between birth and 32 weeks PMA. Hypothesizing, total macronutrient intake before 30 weeks may not reach the threshold to increase concurrent IGF-1 levels, but it might stimulate the IGF-1 axis to mature more rapidly and in this way cause a more rapid increase in IGF-1 levels over time.

### 4.4. Strengths and Limitations

This is the first study to evaluate the contribution of all macronutrients in relation to circulatory IGF-1 levels in preterm infants from birth until 36 weeks of gestation. In addition, to the best of our knowledge, the proportion of parenteral nutrition has not been investigated previously in relation to IGF-1 levels. In line with previous research, our results demonstrate a slow increase in IGF-1 levels in the first weeks of life [4]. However, our results failed to support previous findings on nutrient intake per kg body weight. As previously mentioned, our population had little variation in nutrient intake per kg body weight. This could potentially explain the lack of statistically significant findings. In addition, despite the considerable overall sample size, this study had a relatively small sample size per week. This was due to the low sample frequency (on alternating weeks) and the relatively small number of extremely preterm infants, which resulted in a sample size ranging from 1 infant at 26 weeks PMA to 33 infants at 33 weeks PMA. This may have contributed to our findings. Moreover, it is important to note that this was an exploratory observational study. Therefore, the findings should be interpreted with caution and strong conclusions on potential causative relationships cannot be made.

## 5. Conclusions

Our findings further illustrate the complex association of maturation, concurrent comorbidities, and nutrition in relation to IGF-1 levels. The proportion of parenteral nutrition was found to be a negative predictor of IGF-1 levels, affirming the importance of stimulating enteral nutrition and limiting parenteral nutrition as much as possible in clinical practice. Our findings point towards a potential time frame in which nutrition is unable to impact IGF-1 levels. Future research should aim to narrow down this time frame and to gain more insight into factors enhancing or decreasing the response of IGF-1 to nutrition, e.g., age and inflammatory state, to align nutritional interventions accordingly.

## Figures and Tables

**Table 1:** Reference values used for the nutritional composition of human milk per 100 mL.

Variables	OMM	OMM + BMF (4.4g/100 mL)	DHM	DHM + BMF
Energy (kcal)	68.5	83.8	60	75
Protein (g)	1.5	2.6	0.8	1.9
Protein/energy ratio (g/100 kcal)	2.2/100		1.3/100	
Carbohydrates (g)	7.3	10.0	7.5	10.2
Fat (g)	3.3	3.3	2.9	2.9

BMF: Breast milk fortifier, DHM: donor human milk, OMM: own mother’s milk.

**Table 2:** Sample size for IGF-1 analyses per postmenstrual age.

PMA	24	25	26	27	28	29	30	31	32	33	34	35	36
N Total	2	3	7	17	21	33	30	34	25	33	25	26	29
N Postnatal	0	0	1	3	6	12	16	22	25	33	25	26	29

N Total reflects the total number of samples taken. N Postnatal reflects the number of samples excluding umbilical cord blood.

**Table 3:** Characteristics of the study population.

Variables	(*n* = 87)
Gender, *n* (%)	
Male	44 (50.6)
Female	43 (49.4)
Ethnicity, *n* (%)	
White	66 (75.9)
Other	21 (24.1)
Gestational age (weeks), mean (SD)	29.0 (1.8)
Extremely preterm, *n* (%)	25 (28.7)
Very preterm, *n* (%)	62 (71.3)
Birthweight (g), mean (SD)	1210 (216)
Birthweight SDS, mean (SD)	0.0 (0.7)
Birthweight SDS < −1.3, *n* (%)	3 (3.4)
BPD, *n* (%)	30 (34.5)
NEC, *n* (%)	8 (9.2)
LOS, *n* (%)	30 (34.5)
PDA, *n* (%)	
Hemodynamically Insignificant PDA	11 (12.6)
Hemodynamically Significant PDA	8 (9.2)
ROP, *n* (%)	
ROP stage I	4 (4.6)
ROP stage III	1 (1.1)
IVH, *n* (%)	
IVH grade I	8 (9.2)
IVH grade II	11 (12.6)
IVH grade III	4 (4.6)

BPD: Bronchopulmonary dysplasia, IVH: intraventricular hemorrhage, LOS: Late-onset sepsis; NEC: Necrotizing enterocolitis; PDA: patent ductus arteriosus, ROP: retinopathy of prematurity.

**Table 4:** Regression analyses of parenteral nutrition as a predictor of IGF-1 levels at 2 weeks postnatal age.

Variables	B (SE)	β	*p*-Value
Included variables			
Constant	1.482 (1.359)		0.281
Percentage parenteral intake on day 8	−0.027 (0.011)	−0.234	0.019
Weight on day 8 (grams)	0.004 (0.001)	0.478	<0.001
BPD	−1.134 (0.516)	−0.233	0.032
Hemodynamic significant PDA	−1.350 (0.793)	−0.159	0.095

R² = 0.574, *p* < 0.001; percentage parenteral intake: $\frac{\mathrm{parenteral}\;\mathrm{energy}\;\mathrm{intake}}{\mathrm{total}\;\mathrm{energy}\;\mathrm{intake}}$, BPD: bronchopulmonary dysplasia, PDA: persistent ductus arteriosus, IGF-1: insulin-like growth factor 1.

**Table 5:** Regression analyses of intake as a predictor of IGF-1 levels at 30 weeks postmenstrual age.

Variables	Model R²	Model *p*-Value	B (SE)	β	*p*-Value
Energy intake model 1:	0.605	0.006			
Constant			11.8 (6.7)		0.106
Energy intake (kcal/day)			0.05 (0.02)	0.6	0.015
Gestational age (weeks)			−0.6 (0.2)	−0.5	0.029
Energy intake model 2:	0.640	0.014			
Constant			9.4 (7.1)		0.215
Energy intake (kcal/day)			0.03 (0.03)	0.3	0.395
Gestational age (weeks)			−0.5 (0.2)	−0.4	0.073
Weight (grams)			0.003 (0.003)	0.36	0.348
Protein intake model 1:	0.578	0.009			
Constant			14.5 (6.7)		0.053
Protein intake (g/day)			1.2 (0.4)	0.6	0.013
Gestational age (weeks)			−0.6 (0.2)	−0.5	0.025
Protein intake model 2:	0.625	0.017			
Constant			10.2 (7.6)		0.209
Protein intake (g/day)			0.5 (0.8)	0.2	0.561
Gestational age (weeks)			−0.5 (0.2)	−0.4	0.089
Weight (grams)			0.004 (0.003)	0.4	0.289
Carbohydrate intake model 1:	0.593	0.014			
Constant			9.1 (7.8)		0.268
Carbohydrate intake (g/day)			0.3 (0.1)	0.6	0.022
Gestational age (weeks)			−0.4 (0.3)	−0.4	0.111
Carbohydrate intake model 2:	0.690	0.007			
Constant			5.5 (6.9)		0.444
Carbohydrate intake (g/day)			0.2 (0.1)	0.3	0.144
Gestational age (weeks)			−0.4 (0.2)	−0.3	0.113
Weight (grams)			0.004 (0.002)	0.5	0.052
Fat intake model 1:	0.581	0.008			
Constant			12.3 (6.9)		0.102
Fat intake (g/day)			1.1 (0.4)	0.6	0.012
Gestational age (weeks)			−0.6 (0.2)	−0.5	0.034
Fat intake model 2:	0.631	0.015			
Constant			9.4 (7.2)		0.225
Fat intake (g/day)			0.5 (0.7)	0.3	0.494
Gestational age (weeks)			−0.5 (0.3)	−0.4	0.083
Weight (grams)			0.003 (0.003)	0.4	0.273

IGF-1: Insulin-like growth factor 1.

## Abbreviations

IGF-1: insulin-like growth factor 1, PMA: postmenstrual age, NICU: neonatal intensive care unit, SDS: standard deviation score, BPD: bronchopulmonary dysplasia, NEC: necrotizing enterocolitis, LOS: late onset sepsis, IVH: intraventricular hemorrhage, ROP: retinopathy of prematurity, PDA: patent ductus arteriosus

## References

[1] Larnkjaer A., Molgaard C., Michaelsen K.F. (2012). Early nutrition impact on the insulin-like growth factor axis and later health consequences. Curr. Opin. Clin. Nutr. Metab. Care.

[2] Yumani D.F., Lafeber H.N., van Weissenbruch M.M. (2015). Dietary proteins and IGF I levels in preterm infants: Determinants of growth, body composition, and neurodevelopment. Pediatric Res..

[3] Engstrom E., Niklasson A., Wikland K.A., Ewald U., Hellstrom A. (2005). The role of maternal factors, postnatal nutrition, weight gain, and gender in regulation of serum IGF-I among preterm infants. Pediatric Res..

[4] Hansen-Pupp I., Löfqvist C., Polberger S., Niklasson A., Fellman V., Hellström A., Ley D. (2011). Influence of insulin-like growth factor I and nutrition during phases of postnatal growth in very preterm infants. Pediatric Res..

[5] Smith W.J., Underwood L.E., Keyes L., Clemmons D.R. (1997). Use of insulin-like growth factor I (IGF-I) and IGF-binding protein measurements to monitor feeding of premature infants. J. Clin. Endocrinol. Metab..

[6] Giovannucci E., Pollak M., Liu Y., Platz E.A., Majeed N., Rimm E.B., Willett W.C. (2003). Nutritional predictors of insulin-like growth factor I and their relationships to cancer in men. Cancer Epidemiol. Biomark. Prev..

[7] Kaklamani V.G., Linos A., Kaklamani E., Markaki I., Koumantaki Y., Mantzoros C.S. (1999). Dietary fat and carbohydrates are independently associated with circulating insulin-like growth factor 1 and insulin-like growth factor-binding protein 3 concentrations in healthy adults. J. Clin. Oncol..

[8] Norat T., Dossus L., Rinaldi S., Overvad K., Grønbæk H., Tjønneland A., Olsen A., Clavel-Chapelon F., Boutron-Ruault M.C., Boeing H. (2007). Diet, serum insulin-like growth factor-I and IGF-binding protein-3 in European women. Eur. J. Clin. Nutr..

[9] O’Leary M.J., Xue A., Scarlett C.J., Sevette A., Kee A.J., Smith R.C. (2007). Parenteral versus enteral nutrition: Effect on serum cytokines and the hepatic expression of mRNA of suppressor of cytokine signaling proteins, insulin-like growth factor-1 and the growth hormone receptor in rodent sepsis. Crit. Care (Lond. Engl.).

[10] Wojnar M.M., Fan J., Li Y.H., Lang C.H. (1999). Endotoxin-induced changes in IGF-I differ in rats provided enteral vs. parenteral nutrition. Am. J. Physiol..

[11] Cormack B.E., Harding J.E., Miller S.P., Bloomfield F.H. (2019). The Influence of Early Nutrition on Brain Growth and Neurodevelopment in Extremely Preterm Babies: A Narrative Review. Nutrients.

[12] Embleton N.D., Korada M., Wood C.L., Pearce M.S., Swamy R., Cheetham T.D. (2016). Catch-up growth and metabolic outcomes in adolescents born preterm. Arch. Dis. Child..

[13] Lapillonne A., Griffin I.J. (2013). Feeding preterm infants today for later metabolic and cardiovascular outcomes. J. Pediatr..

[14] Fenton T.R., Kim J.H. (2013). A systematic review and meta-analysis to revise the Fenton growth chart for preterm infants. BMC Pediatr..

[15] Boyce C., Watson M., Lazidis G., Reeve S., Dods K., Simmer K., McLeod G. (2016). Preterm human milk composition: A systematic literature review. Br. J. Nutr..

[16] Gidrewicz D.A., Fenton T.R. (2014). A systematic review and meta-analysis of the nutrient content of preterm and term breast milk. BMC Pediatr..

[17] Jobe A.H., Bancalari E. (2001). Bronchopulmonary dysplasia. Am. J. Respir. Crit. Care Med..

[18] Kliegman R.M., Walsh M.C. (1987). Neonatal necrotizing enterocolitis: Pathogenesis, classification, and spectrum of illness. Curr. Probl. Pediatr..

[19] Bekhof J., Reitsma J.B., Kok J.H., Van Straaten I.H. (2013). Clinical signs to identify late-onset sepsis in preterm infants. Eur. J. Pediatr..

[20] (2005). International Committee for the Classification of Retinopathy of P. The International Classification of Retinopathy of Prematurity revisited. Arch. Ophthalmol..

[21] Papile L.A., Burstein J., Burstein R., Koffler H. (1978). Incidence and evolution of subependymal and intraventricular hemorrhage: A study of infants with birth weights less than 1500 gm. J Pediatr..

[22] Jain A., Shah P.S. (2015). Diagnosis, Evaluation, and Management of Patent Ductus Arteriosus in Preterm Neonates. JAMA Pediatr..

[23] Bertucci J.I., Blanco A.M., Canosa L.F., Unniappan S. (2017). Direct actions of macronutrient components on goldfish hepatopancreas in vitro to modulate the expression of ghr-I, ghr-II, igf-I and igf-II mRNAs. Gen. Comp. Endocrinol..

[24] Hansen-Pupp I., Hellstrom-Westas L., Cilio C.M., Andersson S., Fellman V., Ley D. (2007). Inflammation at birth and the insulin-like growth factor system in very preterm infants. Acta Paediatr..

[25] MohanKumar K., Namachivayam K., Ho T.T., Torres B.A., Ohls R.K., Maheshwari A. (2017). Cytokines and growth factors in the developing intestine and during necrotizing enterocolitis. Semin. Perinatol..

[26] Alzaree F.A., AbuShady M.M., Atti M.A., Fathy G.A., Galal E.M., Ali A., Elias T.R. (2019). Effect of Early Breast Milk Nutrition on Serum Insulin-Like Growth Factor-1 in Preterm Infants. Open Access Maced. J. Med Sci..

